# Validation of a surgical workspace scale during robot‐assisted surgery

**DOI:** 10.1002/rcs.2482

**Published:** 2022-11-22

**Authors:** Hayder Alhusseinawi, Rikke Haase, Sten Rasmussen, Jørgen B. Jensen, Pernille S. Kingo

**Affiliations:** ^1^ Department of Urology Aalborg University Hospital Aalborg Denmark; ^2^ Department of Clinical Medicine Aalborg University Aalborg Denmark; ^3^ Department of Urology Aarhus University Hospital Aarhus Denmark; ^4^ Department of Clinical Medicine Aarhus University Aarhus Denmark

**Keywords:** computer assisted surgery, kidney, minimal invasive surgery, nephrectomy, robot assisted surgery, surgical rating scale, urology, validation study, workspace

## Abstract

**Background:**

A sufficient surgical workspace is crucial to avoid complications. Within classic laparoscopy, many subjective surgical rating scales (SRSs) have previously been used to evaluate the surgical workspace. This study aimed to validate a modified version of the 5‐point SRS during robot‐assisted radical nephrectomy (RARN).

**Methods:**

Thirty‐two intra‐operative videos of intraperitoneal spaces were recorded from eight patients who underwent RARN. To attain the visualisation of different types of workspaces, we recorded 20 s panoramic videos of different pneumoperitoneum, namely 3, 5, 7 and 12 mmHg. The videos were randomised and presented two times to eight experienced robotic surgeons to evaluate the workspace using our modified 5‐point SRS. Both inter‐and intra‐rater reliabilities were tested.

**Results:**

The results of the validation study showed moderate inter‐rater and good to excellent intra‐rater reliability.

**Conclusion:**

This is a valid tool that can be confidently used by future researchers in the field of robot‐assisted surgery.

## 1 INTRODUCTION

A safe and successful robot‐assisted laparoscopic surgery is dependent on creating a workspace in the abdominal cavity to allow for adequate visualisation and sufficient room for manipulation of laparoscopic instruments during the surgical procedure. This is achieved through the establishment of pneumoperitoneum (PnP) using carbon dioxide. PnP facilitates laparoscopy by expanding the abdominal cavity and suppressing the bowels and viscera, thereby giving the laparoscopic surgeon a good intra‐operative view and unhindered manoeuvrability. During the surgery, the surgeon adjusts the PnP and muscle relaxation status together with the anaesthetist to ensure the best workspace throughout the surgery.

Many subjective surgical rating scales (SRSs) have been designed to evaluate the workspace and different interventions proposed to improve it. Boon and colleagues presented 17 different rating scales in their review, but most of them lack a validation test.^1^

A 5‐point SRS was used in laparoscopic herniotomy, prostatectomy and nephrectomy in two different studies. In these studies it was suggested by the authors that increasing the depth of muscle relaxation and/or increasing PnP and changing the position of the patient or surgeon could improve the workspace.^2^, ^3^

Robot‐assisted surgery (RAS) is rapidly expanding within the surgical field making it possible to perform longer and more complex procedures. The 5‐point SRS originally designed for classical laparoscopic surgery, and the item and adjustment suggested by the 5‐point SRS authors no longer fit the current RAS circumstances. Therefore, it is essential to design a more suited SRS for RAS.

With this study, we aimed to validate a modified 5‐point categorial SRS to be used in future studies where the intervention that affects the workspace is a matter of concern during RAS.

## 2 MATERIAL AND METHODS

### 2.1 Surgical workspace scale

Our workspace scale was re‐designed from the previously described SRSs to fit RAS.^2^, ^3^ The appraisal score used to grade the SRS length proposed by Boon et al. was used to determine the best‐suited surgical workspace scale.^1^ By including five adequately defined items, testing both inter‐and intra‐rater reliability and correlating the workspace to the muscle relaxation and/or PnP status, our scale reached a score of 5/5.

The scale was designed in cooperation with a high‐volume robotic surgeon. We tried to keep the scale as simple as possible, taking the advantages and limitations of robotic surgeries into consideration and ensuring that the scale would be applicable to future studies. The main qualitative topics were kept unchanged, namely the extremely poor, very poor, acceptable, good and optimal conditions adopted from the previously described Leiden SRS scale used in laparoscopic surgery.^3^ The suggested interventions in our study consisted of adjustments for muscle relaxation, PnP, assessment of gas leakage and specific direct guidance of bedside assistants was added by our surgeon (R.H.). Our modified surgical workspace scale is described in Table 1.

**TABLE 1:** The surgical workspace scale

*Extremely poor conditions*: The surgeon is unable to work due to the inability to obtain a visible laparoscopic field because of inadequate muscle relaxation or low intra‐abdominal pressure. Additional muscle relaxants must be given, or intra‐abdominal pressure should be increased *Poor conditions*: There is a visible laparoscopic field, but the surgeon is severely hampered by small room with the hazard of tissue damage. Additional muscle relaxants must be given, or intra‐abdominal pressure should be increased *Acceptable conditions*: There is a wide visible laparoscopic field, but still some interference with the surgeon's work. After **one or two** minor adjustments surgery can be completed *Good conditions*: There is a wide laparoscopic working field, but there is some interference, but no need for adjustments *Optimal conditions*: The laparoscopic working field is optimal, and procedure can be completed without any interference
Minor intervention Asses the possibility of gas leakage Specific direct guidance of assistance Assess relaxation status with anaesthetist

### 2.2 Design and inclusion criteria

The study was designed as a prospective cohort study. The study was conducted at a single tertiary urological cancer centre and included all patients above 18 years of age diagnosed with renal tumours who underwent radical nephrectomy from February 2021 to April 2021. Patients with a body mass index >35 kg/m^2^ and/or a previous history of open abdominal surgery were excluded from the study.

### 2.3 Video recording

Thirty‐two videos from eight patients who met the inclusion criteria were recorded. A 20‐s panoramic video was systematically recorded four times during each surgery. After establishing PnP and placement of ports, the PnP pressure decreased to 3 mmHg in furtherance of the worst possible workspace. The PnP was consecutively raised to 5, 7 and 12 mmHg. A new video was recorded with each new pressure. We aimed to simulate the full range of workspace conditions by changing the PnP level. The recorded videos were then duplicated, randomised and embedded in an online survey. Eight experienced robotic surgeons (all consultants in urology and sub‐specialised in onco‐urology) from two high‐volume centres assessed the videos using the surgical workspace scale through an online survey. An example of the video recordings can be seen on https://vimeo.com/660265156.

### 2.4 Statistics

For the study statistics, StataCorp. 2021, Stata Statistical Software, ver. 17. College Station, TX: StataCorp LLC was used.

The precision approach was used to determine the sample size.^4^, ^5^ Using the sample size determination approach described by Bonett^6^ and assuming an inter‐class correlation coefficient (ICC) = 0.80, eight raters and a 95% confidence interval (CI) around ICC of width (0.2) gave a sample size of *n* = 25 records where each record was regarded as an individual subject (seven patients). Given a predicted dropout of 10% due to possible damage to recorded videos or poor‐quality recordings, eight patients were included (32 records).

Although the equivalence of results from the ICC and weighted kappa tests has been previously described,^7^ the research group considered running both statistical methods.

To assess the inter‐rater reliability, we used a two‐way random‐effect model with absolute agreement ICC and a Fleiss weighted (quadratic) kappa. To test intra‐rater reliability (test‐retest), we used a two‐way mixed‐effect model with absolute agreement ICC.

The following classifications have been suggested by Koo et al. for assessing how good the strength of agreement is based on the value of the ICC. Values <0.5 indicate poor reliability, values between 0.5 and 0.75 indicate moderate reliability, values between 0.75 and 0.9 indicate good reliability and values >0.90 indicate excellent reliability.^8^

### 2.5 Ethics

This study was conducted according to the national guidelines on reporting clinical trials and was approved by the institutional review board and the research ethical committee in the Northern Region of Jutland (Protocol No. N‐20200078, approval date 08 December 2020). All patients received both oral and written information about the study and signed consent was obtained.

## 3 RESULTS

Patient demographics are shown in Table 2.

**TABLE 2:** Demographics of eight patients who underwent RARN due to renal cancer

Age	58 (49–64)
Sex	
Male	5 (62.5%)
Female	3 (37.5%)
BMI	24.3 (±4)
DM	3 (37%)
Hypertension	2 (25%)

*Note*: Data are mean (SD), *n* (%), or median (IQR).

Abbreviations: BMI, body mass index; DM, diabetes mellitus; RARN, robot‐assisted radical nephrectomy.

One video record was excluded due to technical damage.

The pooled inter‐rater agreement between surgeons showed moderate reliability with an ICC of 0.74 95% CI (0.66–81) with a slight difference between the first and second evaluations. Results from the weighted Fliess kappa test are shown in Table 3.

**TABLE 3:** Inter‐rater reliability of eight surgeons assessing workspace of 31 recorded videos

First evaluation		
Inter‐rater reliability tested by Fleiss kappa	0.75	95% CI (0.63–0.87)
Inter‐rater reliability tested by ICC	0.76	95% CI (0.64–0.85)
Second evaluation		
Inter‐rater reliability tested by Fleiss kappa	0.72	95% CI (0.60–0.84)
Inter‐rater reliability tested by ICC	0.73	95% CI (0.62–0.83)

Abbreviations: CI, confidence interval; ICC, intraclass correlation coefficient.

The intra‐rater reliability (test‐retest) of eight surgeons shows good to excellent reliability. The ICC and 95% CI ranged between 0.80 (0.62–0.89) and 0.98 (0.97–0.99) and the pooled ICC was 0.89 (0.86–91).

The ICC and CI of the surgical workspace scale for all surgeons are shown in Table 4. All surgeons used the full‐scale range from 1 to 5 at almost the same frequency; however, they used item 3 more frequently than the other scale items (30%).

**TABLE 4:** Intra‐rater reliability of surgical workspace scale for eight surgeons

Raters (surgeons) number	ICC	95% CI
1	0.90	0.81–0.95
2	0.92	0.84–0.96
3	0.83	0.62–0.92
4	0.90	0.81–0.95
5	0.82	0.64–0.91
6	0.80	0.62–0.89
7	0.88	0.77–0.94
8	0.98	0.97–0.99
The pooled reliability	0.89	0.86–0.91

Abbreviations: CI, confidence interval; ICC, intraclass correlation coefficient.

The surgeons changed their rating approximately seven times between the first and second evaluations. Among all surgeons a total of 53 changes were made.

## 4 DISCUSSION

This is the first prospective observational study that aimed to validate the surgical workspace scale to be used for robotic surgery studies, especially where the assessment of the surgical workspace is the outcome of focus during the implementation of new techniques or measures. This study showed moderate inter‐rater and good to excellent intra‐rater reliability.

Throughout the years, many different SRSs have been designed. As previously mentioned, most of these scales lacked validation.^1^

Martini et al. developed their 5‐point Leiden‐surgical rating scale for laparoscopic urologic surgery.^3^ The scale items are well‐described and incorporate visibility of critical structures, working space and muscle contractions as determinants of the surgical working field. They tested the workspace to compare deep versus moderate muscle relaxation, focussing on muscle contraction and relaxation as a parameter for scoring without considering PnP. The scale was tested for inter‐rater reliability by comparing the rating score of the operating surgeon with the rating score of 12 anaesthesiologists, finding poor agreement. The authors attributed the results to anaesthesiologists being less capable of evaluating surgical conditions during laparoscopy from video images and hence deriving insufficient information from these images regarding the working conditions of the surgeon. Hence, a 30‐s video image does not provide sufficient input to assess the quality of surgical conditions in non‐surgically skilled personnel.

Furthermore, in a study by Nervil et al. using another design for a 5‐point SRS, the researchers assessed both inter‐and intra‐rater reliabilities.^2^ The studies showed fair inter‐rater agreement but an excellent intra‐rater agreement.

The study has several strengths, including the use of the full range of the scale (1–5) during the evaluation process. The detailed description of scale points with flexibilities of possible major and minor interventions decreased the variability between the raters, which could be the reason why our scale has higher inter‐rater reliability compared to previous similar studies. We recruited eight robotic surgeons within onco‐urology from two high‐volume robotic centres. Every surgeon had performed at least 200 robotic surgeries before the time of recruitment to add to the solidity and credibility of our results.

The major limitation of this study is a subjective evaluation of the workspace that is difficult to prevent. The definition of acceptable workspace could differ for different surgeons. While some surgeons evaluated the low Pnp (7 mmHg) as an acceptable workspace other surgeons scored both low and ultra‐low Pnp (5 mmHg) as acceptable. This is well presented in our data as item 3 of the scale that refer to the acceptable workspace was the most frequently used item. The other limitation reported by some of the surgeons who evaluated the video was the absence of surgical instruments and tissue handling, which led to the inaccurate evaluation of some videos. Therefore, when the surgical workspace scale is used in future studies, we recommend using the scale multiple times during the surgery in the predefined steps of the surgical procedure, which could be challenging for a surgeon in terms of workspace.

## 5 CONCLUSION

The modified workspace scale described here shows moderate inter‐rater and good to excellent intra‐rater reliability. Therefore, it is a valid tool that can be confidently used by future researchers in the field of RAS.

## CONFLICT OF INTEREST

The authors have no conflicts of interest to declare that are relevant to the content of this article.

## Data Availability

The data that support the findings of this study are available from the corresponding author upon reasonable request.
